# Delayed Recognition of Hemophagocytic Lymphohistiocytosis in a Child With Refractory Fever: A Case From Pakistan

**DOI:** 10.1002/ccr3.73507

**Published:** 2026-09-15

**Authors:** Muhammad Taaha Siddiqui, Nawal Fatima, Khola Qazi, Karan Chaman Lal, Md. Faisal Ahmed

**Affiliations:** ^1^ Department of Medicine Continental Medical College Lahore Punjab Pakistan; ^2^ Department of Medicine Jinnah Sindh Medical University Karachi Sindh Pakistan; ^3^ Department of Medicine Liaquat University of Medical & Health Sciences Jamshoro Sindh Pakistan; ^4^ Bangladesh Institute of Innovative Health Research Mirpur Dhaka Bangladesh

**Keywords:** cytopenia, fever of unknown origin, hemophagocytic lymphohistiocytosis, hyperferritinemia, hyperinflammation, pediatric

## Abstract

Hemophagocytic lymphohistiocytosis (HLH) is a life‐threatening, exceedingly rare hyperinflammatory syndrome that typically presents with nonspecific symptoms such as fever and cytopenia. An early diagnosis of HLH is a significant clinical challenge, especially in children with presentations mimicking common infectious diseases. A 9‐year‐old girl came with a 2‐week history of intractable high‐grade fever, cough, and generalized weakness, unresponsive to broad‐spectrum antibiotics. Physical examination showed hepatosplenomegaly and pallor. Laboratory tests showed pancytopenia, significantly elevated serum ferritin (1144 ng/mL), hypertriglyceridemia (349 mg/dL), and hypofibrinogenemia (107 mg/dL). Imaging showed hepatosplenomegaly and mild brain atrophic changes. Despite treatment with multiple antimicrobial therapies, the fever persisted, and thus, suspicion of HLH arose. She met five out of eight HLH‐2004 diagnostic criteria, and on this basis, a presumptive diagnosis was established. Treatment consisted of supportive care, antipyretics, transfusions, and continued observation. Given the complexity of her condition and failure to achieve sufficient clinical improvement, she was subsequently transferred to a higher care facility for specialist management. This case underscores the need to keep HLH in mind in children with chronic, treatment‐resistant febrile illness, particularly in the setting of cytopenia and organomegaly. Importantly, this case illustrates that even when basic laboratory investigations are readily available at tertiary referral centers, delayed clinical recognition of HLH remains the primary diagnostic challenge in Pakistan. The critical gap is not always the absence of tests but the absence of HLH in the differential diagnosis. Heightened physician awareness, early clinical suspicion, and timely referral are essential to improving outcomes in low and middle‐income country settings.

## Key Clinical Message

Hemophagocytic lymphohistiocytosis (HLH) is a critical diagnostic challenge in pediatric patients with refractory fever. In Pakistan, the primary hurdle is delayed clinical recognition rather than testing availability. Improving physician awareness and maintaining early suspicion are the most essential tools for ensuring timely diagnosis and life‐saving intervention.

## 1 Introduction

Hemophagocytic lymphohistiocytosis (HLH) is a hyperinflammatory, hyperferritinemic syndrome characterized by uncontrolled activation of cytotoxic T lymphocytes and macrophages, leading to excessive cytokine release [1]. Hemophagocytosis by activated histiocytes initiates a self‐sustaining inflammatory cascade that ultimately leads to multiorgan dysfunction [2]. HLH is classified as either primary (genetic) or secondary (acquired). Primary HLH typically follows an autosomal recessive inheritance pattern and is associated with mutations in PRF1, UNC13D, STX11, and STXBP2 [3]. Secondary HLH arises due to infections—most commonly Epstein–Barr virus and Dengue virus—malignancies, or autoimmune disorders, and is associated with a mortality rate exceeding 40% if left untreated [4].

Diagnosis of HLH requires fulfillment of at least five out of eight HLH‐2004 criteria: (1) fever of 38.3°C or higher; (2) splenomegaly; (3) cytopenia affecting at least two blood cell lineages, defined as hemoglobin below 9 g/dL, platelets under 100,000 per microliter, or neutrophils below 1000 per microliter; (4) hypertriglyceridemia (265 mg/dL or more) and/or hypofibrinogenemia (150 mg/dL or less); (5) evidence of hemophagocytosis in bone marrow, spleen, lymph nodes, or liver; (6) low or absent natural killer cell activity; (7) ferritin of 500 ng/mL or higher; and (8) elevated soluble CD25 (sIL‐2Rα) of 2400 U/mL or more [1]. A molecular diagnosis consistent with HLH also confirms the disease. Management focuses on controlling hyperinflammation, typically initiated with dexamethasone, cyclosporine, and etoposide, as outlined in the HLH‐94 and HLH‐2004 protocols. Hematopoietic stem cell transplantation is recommended for primary HLH [1]. The following report presents the case of a 9‐year‐old female child who was misdiagnosed as suffering from enteric fever. In this condition, the patient met the criteria of HLH 1, 2, 3, 4, and 7.

## 2 Case History/Examination

A 9‐year‐old female child weighing 25 kg presented with high‐grade fever, 104°F, persisting for more than 2 weeks, associated with cough, chest congestion, poor oral intake, fatigue, and pallor. The acute presentation came following a relapsing period of intermittent fever, which lasted about 1–2 months before admission, indicating that she had a chronic inflammatory process going on before the patient's clinical status deteriorated and required hospitalization. She was on empiric intravenous antibiotics like meropenem, piperacillin‐tazobactam, linezolid, and vancomycin for 2 weeks based on the provisional diagnosis of complicated enteric fever. However, despite the antibiotic treatment, the fever continued, which made us broaden the diagnosis.

On examination, she was conscious but irritable with mild pedal edema. On abdominal examination, there was a liver span of 3–4 cm and a spleen span of 4–5 cm below the costal margin. Bilateral crepitations were detected on chest auscultation without evidence of pleural effusion. Neurological examination remained unremarkable throughout the admission. Serial ultrasound scans confirmed hepatosplenomegaly with mild pericholecystic and pelvic free fluid.

## 3 Methods

### 3.1 Differential Diagnosis and Investigations

Considering the presence of fever for a considerable time period, endemic exposure, and hepatosplenomegaly, complicated enteric fever constituted the primary diagnosis. Nevertheless, negativity on blood culture and non‐response to broad‐spectrum antibiotics cast doubts on the diagnosis. Hematologic malignancies were suspected based on organomegaly and cytopenia, but in the absence of blasts on peripheral smear and the inability to perform bone marrow examination, the possibility could not be confirmed. The condition of Macrophage Activation Syndrome (MAS) was one of the considerations, but in the absence of rash, arthritis, or autoimmune markers, the probability was low. Tuberculosis and brucellosis were ruled out by serological tests and clinical findings. HLH could not be definitively ruled out without genetic studies. The probability of autoimmune disorders such as SLE was lower, considering the negative result on the antinuclear antibody test. This finding suggests that HLH is likely to be a secondary disease condition resulting from either a viral infection or a hematologic malignancy. According to HLH‐2004 criteria, the patient met five out of eight main criteria (Criteria 1, 2, 3, 4, and 7) and is therefore diagnosed as having HLH (Table 1).

**TABLE 1:** Laboratory investigations at initial presentation to the tertiary care facility (Hospital Day 1).

Parameter	Result	Normal range	Interpretation
Hemoglobin (Hb)	8.4 g/dL	11.5–15.5 g/dL	Severe anemia
Hematocrit (HCT)	26.2%	35.0%–45.0%	Low
RBC count	3.93 × 10^12^/L	4.0–5.2 × 10^12^/L	Mildly reduced
MCV	66.7 fL	77–95 fL	Microcytic
MCH	21.4 pg	25–33 pg	Hypochromic
MCHC	32.1 g/dL	31–37 g/dL	Normal
RDW	22.3%	12.1%–16.9%	High (anisocytosis)
WBC count	8.5 × 10^9^/L	5.0–13.0 × 10^9^/L	Normal
Neutrophils	7.3%	40%–75%	Severe neutropenia
Lymphocytes	39.2%	20%–45%	Normal
Monocytes	53.4%	2%–10%	Marked monocytosis
Eosinophils	0.0%	1%–6%	Absent
Basophils	0.1%	0%–1%	Normal
Platelets^a^	114 × 10^9^/L	180–400 × 10^9^/L	Thrombocytopenia
Serum triglyceride	349 mg/dL	< 150 mg/dL	High
Plasma fibrinogen levels	107 mg/dL	156–400 mg/dL	Low
Serum ferritin	1144.0 ng/mL	7–140 ng/mL	High
Serum albumin	2.3 g/dL	3.5–5.0 g/dL	Low
ALT	70 U/L	Up to 40 U/L	Elevated (hepatic involvement)
ALP	239 U/L	Up to 400 U/L	Normal
AST	74 U/L	Up to 50 U/L	Elevated
Total bilirubin	1.1 mg/dL	0.3–1.0 mg/dL	Elevated
ANA	Negative	—	—
ASMA	Negative	—	—
AMA	Negative	—	—
Brucellosis	Negative	—	—
Tuberculosis	Negative	—	—

Abbreviations: ALP, alkaline phosphatase; ALT, alanine aminotransferase; AMA, anti‐mitochondrial antibodies; ANA, anti‐nuclear antibodies; ASMA, anti‐smooth muscle antibodies; AST, aspartate aminotransferase; Hb, hemoglobin; HCT, hematocrit; MCH, mean corpuscular hemoglobin; MCHC, mean corpuscular hemoglobin concentration; MCV, mean corpuscular volume; RBC, red blood cell; RDW, red cell distribution width; WBC, white blood cell.

^a^ Platelet count shown reflects the Day‐1 presentation value. Serial monitoring during admission showed a subsequent downward trend, with a nadir of 25,000/μL, meeting the HLH‐2004 thrombocytopenia threshold (< 100 × 10^9^/L). See Discussion for full serial values.

### 3.2 Serology

Hematological derangements were significant in laboratory investigations, i.e., anemia, thrombocytopenia, and neutropenia, along with increased inflammatory markers consistent with systemic hyperinflammation. Hyponatremia, along with a slight elevation of liver enzymes were also noted.

The autoimmune serology results were as follows: ANA negative with a titrated endpoint of 1/80, ASMA negative, and AMA negative. Brucella serology was negative. These results ruled out SLE, autoimmune hepatitis, primary biliary cirrhosis, and other connective tissue diseases.

One Paeds Plus bottle exhibited positive growth for non‐aureus staphylococcus in blood cultures. Microscopic examination revealed Gram‐negative bacilli, but there was no growth in cultures, which may be attributed to previous antibiotic use or the fastidious nature of the organism. It was considered clinically irrelevant, making infection an unlikely cause for this condition.

Regarding the inflammatory markers, there were some values found to be elevated in the blood work, as follows: Serum ferritin: 1144.0 ng/mL (Reference: 7–140 ng/mL, age 6 months–15 years); > 500 ng/mL is considered the HLH cut‐off. Serum triglyceride levels: 349 mg/dL (Reference: < 150 mg/dL); > 265 mg/dL is considered the HLH cut‐off. Plasma fibrinogen levels: 107 mg/dL (Reference: 156–400 mg/dL); < 150 mg/dL indicates hypofibrinogenemia.

### 3.3 Abdominal Ultrasound

Serial abdominal ultrasounds revealed the presence of hepatosplenomegaly with mild pericholecystic and pelvic fluid, thus giving radiological proof of organomegaly consistent with the criteria for HLH.

### 3.4 Chest X‐Ray

**FIGURE 1:**
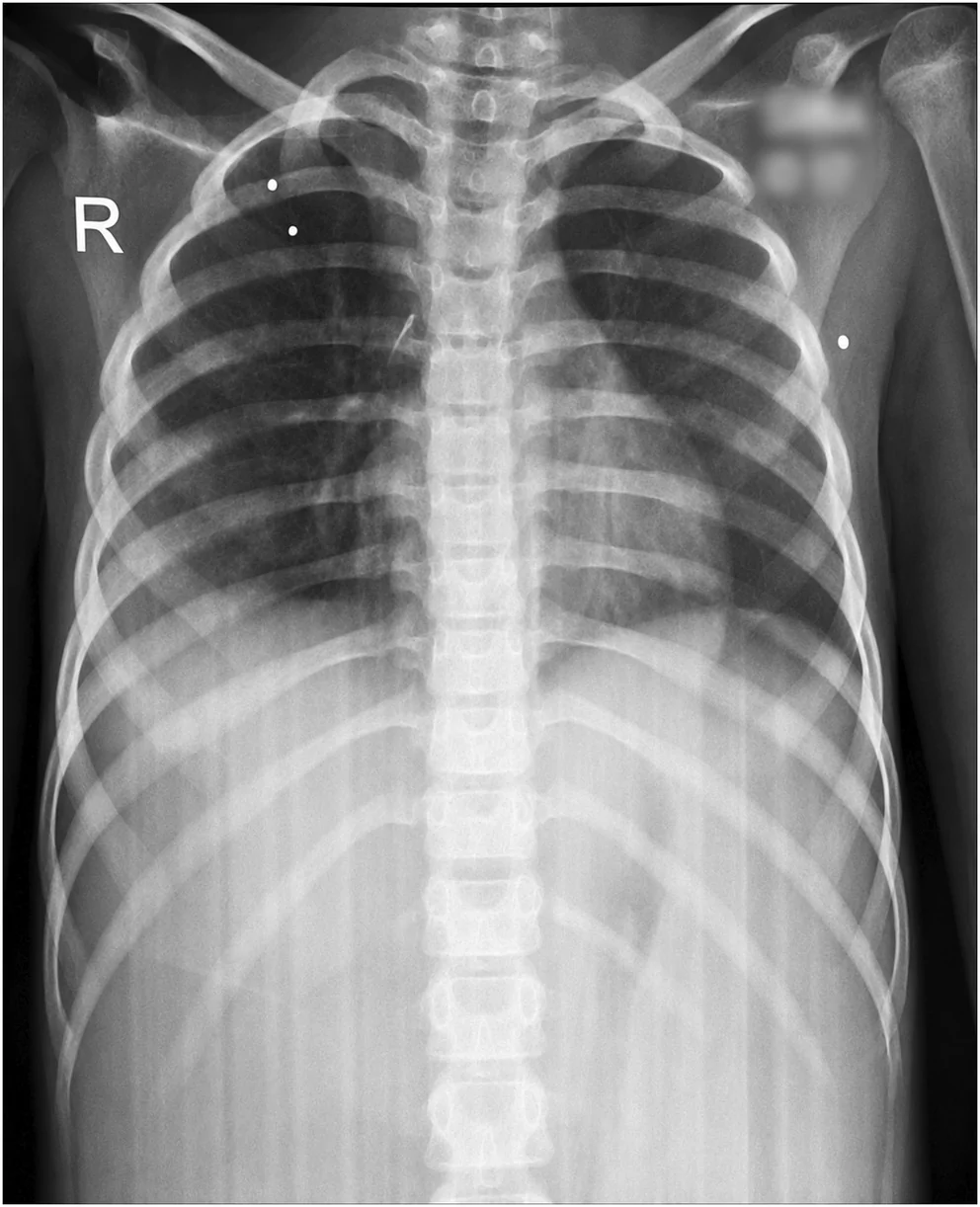
De‐identified chest X‐ray (PA view) obtained at initial presentation, showing normal lung fields, absence of cardiomegaly, and absence of pleural effusion or mediastinal widening. These findings exclude pulmonary infection or malignancy as a cause of the patient's chest congestion, supporting the systemic, non‐pulmonary nature of the underlying hyperinflammatory process. Patient‐identifying information has been removed from the image before submission.

Figure 1 De‐identified chest X‐ray (PA view) obtained at initial presentation, showing normal lung fields, absence of cardiomegaly, and absence of pleural effusion or mediastinal widening (Figure 1). These findings exclude pulmonary infection or malignancy as a cause of the patient's chest congestion, supporting the systemic, non‐pulmonary nature of the underlying hyperinflammatory process. Patient‐identifying information has been removed from the image before submission.

### 3.5 MRI Brain

A contrast‐enhanced MRI of the brain was performed in view of the patient's fever and irritability. Findings demonstrated mild increased intra‐ and extra‐axial CSF spaces with prominent ventricles and sulci, representing brain atrophic changes. Prominent folia were also identified involving the cerebellum. No pathological post‐contrast enhancement was noted. There was no definite evidence of intracranial bleed, gross area of infarction, or mass lesion. Cerebral hemispheres showed a normal sulci and gyri pattern. Ventricles, CSF cisterns, and white matter appeared normal. The basal ganglia, thalami, and brain stem appeared normal. Both cerebellar hemispheres and vermis were normal, and the vestibulo‐cochlear nerve complexes, sella, pituitary gland, and cavernous sinuses were unremarkable. Notably, mucoperiosteal thickening was identified involving the bilateral maxillary sinuses, ethmoid air cells, and sphenoid sinus, representing sinus inflammatory disease. The overall impression confirmed mild brain atrophic changes with the remainder of the scan appearing unremarkable, necessitating clinical and laboratory correlation.

### 3.6 Treatment

The management was largely supportive in the face of emerging diagnostic findings. Empirical broad‐spectrum antibiotic treatment for over 3 weeks with meropenem, piperacillin‐tazobactam, linezolid, and vancomycin was done. Despite prolonged antibiotic treatment, the fever persisted. An albumin infusion was done to correct hypoalbuminemia. Antipyretic medications were administered every 4 h, along with physical cooling measures. However, a persistent fever above 38.5°C persisted despite this. Packed red blood cell transfusions were performed for anemia, and IV fluids were administered throughout the admission.

Notably, immunosuppression treatment based on HLH, that is, the HLH‐94/HLH‐2004 protocol involving dexamethasone, etoposide, and cyclosporine was not started. This is a major deficit in management since early immunosuppression leads to significantly higher survival rates in proven or suspected cases of HLH [5, 6]. This failure to provide HLH treatment is mainly attributed to late diagnosis.

## 4 Conclusion and Results

Although the patient received vigorous supportive care, her condition did not improve significantly, and she was transferred to a tertiary care center for further evaluation. HLH‐specific therapy was not provided in this case, which could be considered a crucial management gap. From this case, we can infer that the main problem with HLH identification is not only the lack of diagnostic tests but also the failure to consider HLH in the differential diagnosis. It is important to increase awareness about HLH among medical professionals of all tiers, make referral guidelines, and add HLH to the list of differentials in cases of persistent fever in children in countries like Pakistan and others with similar economic situations. HLH‐directed treatment with the HLH‐94/HLH‐2004 regimen, including dexamethasone, etoposide, and cyclosporine, was not administered during this admission, which represents another major management gap. HLH‐directed treatment can dramatically improve the survival rates in cases of confirmed or suspected HLH [5, 6].

## 5 Discussion

In this case, a 9‐year‐old girl presented with chronic high‐grade fever, hepatosplenomegaly, cytopenia, hyperferritinemia, hypertriglyceridemia, and decreased fibrinogen. These features fulfill at least five of the eight HLH‐2004 diagnostic criteria [1].

Hemophagocytic lymphohistiocytosis is extremely rare, with a global incidence of approximately 1.2 cases per million children per year. This estimate is likely an underrepresentation, as the disease is frequently missed or misdiagnosed due to diagnostic delays and challenges in clinical settings. The rarity and non‐specific features of HLH make it one of the most elusive hyperinflammatory syndromes in children. Its symptoms resemble those of common conditions such as sepsis, enteric fever, viral infections, hematologic malignancies, and autoimmune disorders, complicating early diagnosis. Fever, present in nearly all HLH cases, is difficult to distinguish from common infections without specific testing. Hepatosplenomegaly, cytopenia, and elevated inflammatory markers—hallmarks of HLH—also occur in tuberculosis, lymphoma, juvenile arthritis, and severe sepsis. Such a combination forms one of the main reasons behind the misdiagnosis of HLH. This patient was initially treated as a case of complicated enteric fever, which could be possible due to the nature of his symptoms. However, as the disease did not subside even after almost 3 weeks of treatment with broad‐spectrum antibiotics like Meropenem, Tizano, Colomycin, and Linezolid, the need to alter the diagnosis arose. In the context of adequate antibiotic coverage, refractory fever in children must continually raise suspicion of hyperinflammatory syndromes such as HLH [7, 8]. In the current case, prolonged fever over 38.5°C, despite antipyretics administered every 4 h and sponging, was one of the most recalcitrant clinical features.

Hepatosplenomegaly was confirmed by clinical examination and radiological assessment, with the spleen measuring 15.9 cm. Organ enlargement and hematologic abnormalities, including hemoglobin less than 9 g/dL and a platelet nadir of 25,000/μL recorded later in the admission (Table 1 reflects the Day‐1 value of 114 × 10^9^/L), fulfilled the cytopenia criterion for HLH. The absolute neutrophil count was less than 1.0 × 10^9^/L, which meets the requirements for cytopenia in at least two hematopoietic lineages [1, 9].

Serum ferritin was 1144 ng/mL, well above the HLH‐2004 diagnostic threshold of ≥ 500 ng/mL and the reference range of 7–140 ng/mL for this age group. Triglycerides were 349 mg/dL, classified as High per NCEP ATP III guidelines and exceeding the HLH threshold of ≥ 265 mg/dL. Plasma fibrinogen was 107 mg/dL, below the normal range of 156–400 mg/dL and meeting the HLH criterion for hypofibrinogenemia (≤ 150 mg/dL); this qualifies for HLH criteria for metabolic dysregulation [1, 5]. The findings indicate a cytokine‐mediated, not infectious, process. The autoimmune serology panel showed ANA negative (titer 1/80), ASMA negative, and AMA negative. Brucella serologies were negative, which excluded autoimmune and other infectious disorders [8, 9]. Blood culture grew only in a single Paeds Plus bottle, Staphylococcus Species (Not Aureus); the lab noted this isolate was likely not clinically significant, further suggesting a non‐infectious diagnosis.

The patient had no focal neurological deficits; however, MRI brain with contrast revealed slightly increased intra‐ and extra‐axial CSF spaces with prominent ventricles and sulci representing atrophic changes in the brain and mucoperiosteal thickening involving the bilateral maxillary sinuses, ethmoid air cells, and sphenoid sinus, representing sinus inflammatory disease. Pathological post‐contrast enhancement, intracranial bleed, infarction, or mass lesion were not identified. These findings could indicate central nervous system involvement, as it is a known complication in 20%–25% of HLH cases [10]. The elevation of liver enzymes and requirement for albumin transfusion suggested hepatic involvement. Despite the lack of assessment of bone marrow biopsy, natural killer (NK) cell activity, and soluble interleukin‐2 receptor levels, the clinical diagnosis of HLH remains justified with the available data [1, 4].

The treatment of HLH is time‐sensitive and fundamentally distinct from the management of infectious febrile illnesses, which is why diagnostic delay carries such grave consequences. The backbone of HLH therapy includes the HLH‐94/HLH‐2004 regimen that utilizes etoposide and dexamethasone for the initiation of treatment, with the inclusion of cyclosporine based on the newer 2004 protocol [1, 6]. Etroposide suppresses the immune system hyperactivity by attacking fast‐dividing cells like lymphocytes and macrophages; on the other hand, dexamethasone is useful for broad immunosuppression and also able to penetrate the BBB to control the neurological symptoms. HSCT is the only curative modality for primary HLH and secondary HLH unresponsive to the initial regimen [3]. For secondary HLH, treatment must additionally address the underlying trigger in parallel with immunosuppressive induction. No HLH‐directed therapies were initiated in this case, which represents a critical management gap. Prompt initiation of etoposide and dexamethasone, even on a presumptive diagnosis, has been shown to significantly reduce mortality and prevent irreversible multiorgan damage [5, 11]. The absence of treatment in this case underscores the real‐world consequences of delayed diagnosis and the importance of empirical HLH therapy when clinical and laboratory findings are sufficiently compelling.

For Pakistan and similar LMICs, this case is especially significant; yet, its ramifications deserve due deliberation. The patient was managed at a tertiary care facility that possessed basic laboratory facilities, such as those for measuring serum ferritin levels, triglycerides, fibrinogen levels, and autoimmune markers. Certain salient findings can be made here. Firstly, no conclusive HLH diagnostic investigation, including bone marrow aspiration, NK cell function test, sIL‐2Rα concentration, and EBV PCR, was performed. Second, the primary barrier in this case was not the lack of laboratory tests but the delayed clinical recognition of HLH as a diagnostic possibility. Even with persistent fever, cytopenia, and organomegaly, this case continued to be diagnosed as enteric fever, highlighting the rare inclusion of HLH in the differential diagnosis in Pakistan. Third, most Pakistani children with similar presentations are managed at district‐level hospitals and smaller facilities where even basic ferritin testing and specialist consultation are unavailable, making this tertiary‐center case unrepresentative of the broader national experience. The principal LMIC challenge, therefore, lies not only in laboratory access but also in the significant gap in HLH awareness among front‐line physicians, the absence of structured referral pathways for hyperinflammatory syndromes, and the lack of region‐specific clinical guidelines that encourage early consideration of HLH in refractory febrile illness. Targeted physician education programs, HLH awareness campaigns, and the integration of HLH into the standard differential diagnosis of treatment‐resistant fever in children are urgently required across Pakistan and similar LMIC settings.

A significant limitation in this case is that an ultrasound report that quantitatively documented the presence of hepatosplenomegaly on first presentation was not available. The organomegaly was assessed clinically, and subsequent ultrasounds were reported qualitatively without giving measurements for the organs involved at each stage. Also, the absence of reported therapy for HLH, including dexamethasone or etoposide, which are standard components of established protocols such as HLH‐94 and HLH‐2004 [1, 6]. Timely therapeutic intervention is critical to prevent irreversible organ damage and improve survival outcomes [5, 11]. However, in many settings, the lack of specific diagnostic tools and targeted therapies can delay appropriate management.

## Author Contributions

**Muhammad Taaha Siddiqui:** writing – review and editing, writing – original draft, conceptualization, investigation, data curation. **Nawal Fatima:** writing – original draft, writing – review and editing, visualization, data curation. **Khola Qazi:** visualization, writing – original draft, writing – review and editing. **Karan Chaman Lal:** writing – original draft, writing – review and editing. **Md. Faisal Ahmed:** writing – review and editing.

## Funding

The authors have nothing to report.

## Ethics Statement

No ethical review was required as the case was obtained with guardian consent.

## Consent

Written informed consent was obtained from the patient's legal guardian for publication of this case report.

## Conflicts of Interest

The authors declare no conflicts of interest.

## Data Availability

The authors have nothing to report.
